# VLSI Implementation of a High-Performance Nonlinear Image Scaling Algorithm

**DOI:** 10.1155/2021/6297856

**Published:** 2021-07-21

**Authors:** Osamah Ibrahim Khalaf, Carlos Andrés Tavera Romero, A. Azhagu Jaisudhan Pazhani, G. Vinuja

**Affiliations:** ^1^Al-Nahrain University, Baghdad, Iraq; ^2^Universidad Santiago de Cali, Cali, Colombia; ^3^Ramco Institute of Technology, Rajapalayam, Tamilnadu, India; ^4^Vins Womens Christian College of Engineering, Kanyakumari, Tamilnadu, India

## Abstract

This study implements the VLSI architecture for nonlinear-based picture scaling that is minimal in complexity and memory efficient. Image scaling is used to increase or decrease the size of an image in order to map the resolution of different devices, particularly cameras and printers. Larger memory and greater power are also necessary to produce high-resolution photographs. As a result, the goal of this project is to create a memory-efficient low-power image scaling methodology based on the effective weighted median interpolation methodology. Prefiltering is employed in linear interpolation scaling methods to improve the visual quality of the scaled image in noisy environments. By decreasing the blurring effect, the prefilter performs smoothing and sharpening processes to produce high-quality scaled images. Despite the fact that prefiltering requires more processing resources, the suggested solution scales via effective weighted median interpolation, which reduces noise intrinsically. As a result, a low-cost VLSI architecture can be created. The results of simulations reveal that the effective weighted median interpolation outperforms other existing approaches.

## 1. Introduction

Digital image interpolation or scaling is an issue that has recently received great attention. Image scaling is a process of resizing a digital image, and it is a nontrivial process that involves a tradeoff between efficiency, smoothness, and sharpness. Nowadays, the image scalar is widely adopted in portable healthcare devices, digital electronic equipment, digital camera, digital photo frame, mobile phone, touch panel computers, etc. It has become a significant trend to design a low-cost, high-quality, and high-performance image scalar by the VLSI technique for multimedia products. As the graphic and video applications of mobile handset devices grow up, the demand and significance of image scaling are more and more outstanding. The image scaling algorithms based on interpolation are basically of two types: linear and nonlinear interpolation methods. The simplest linear interpolation method is a nearest neighbour algorithm which is a low-complexity algorithm, but it results in scaled images with blocking and aliasing artifacts. The most widely used scaling method is bilinear interpolation algorithm by which the target pixel can be obtained by using the linear interpolation model in both horizontal and vertical directions. Another popular polynomial-based method is bicubic interpolation algorithm, which uses an extended cubic model to acquire the target pixel by a 2D regular grid. The nonlinear interpolation methods such as weighted median interpolation, curvature interpolation, bilateral filter, and autoregressive model greatly improve image quality by reducing blocking, aliasing, and blurring effects compared to linear methods.

Many image scaling algorithms have been developed mostly based on interpolation and are edge-oriented. In this section, major scaling algorithms are explained. An edge-oriented area-pixel scaling processor was implemented with low-complexity VLSI architecture [1]. A simple edge-catching technique is adopted to preserve the image edge features. A JPEG edge-oriented area-pixel scaling processor performs scale-up/scale-down transformation by using the area-pixel model instead of the common point model with a simple edge-catching technique to preserve edge features effectively so as to achieve better image quality [2]. The direct implementation of area-pixel scaling requires some extensive floating-point computations so that a suitable approximate low-cost VLSI implementation technique has been used. A novel image zooming algorithm using curvature interpolation was developed. It results in clear images of sharp edges which are already denoised and superior to those obtained from linear methods and PDE-based methods [3]. A real-time FPGA architecture of the extended linear convolution for the image scaling method [4] provides simple hardware architecture design with low computation cost. Compared to the latest bicubic hardware design [5], the architecture saves about 60% of hardware cost.

A low-complexity memory-efficient image scaling processor uses bilinear interpolation with combined sharpening and clamp filters as the prefilter to reduce the blurring and aliasing artifacts [6]. The prefilter is implemented efficiently using T and inverse T models which need two line buffers for processing. Recently, a high-boost filtering-based image scaling algorithm has been developed [7, 8]. In the research environment, improved image processing based on both digital and remote sensing [9] images is analyzed by machine learning techniques [10–13] such as deep network [14], neural network [15, 16], and Markov analysis [17]. Other authors are well-versed in researching profound information to some level and their researches are cited as [18–20]. Wireless sensor network (WSN) [21–28], mobile ad hoc network (MANET) [29–31], web application [32, 33], cryptography security [34, 35], and cloud computing [36] play a significant role in the Internet of Things (IoT) [37, 38]. This efficient IoT network handles a huge volume of data named as big data (BD). This unstructured way of big data information holds huge irrelevancy along with redundant image details which are usually difficult to handle and access. So, the researchers proposed several new BD approaches [39, 40] to acquire the relevant details from the web. By these data, an effective image scaling approach is designed to insist its performance in the VLSI architecture.

In the proposed work, instead of using linear interpolation, a nonlinear method is adopted to enhance the performance of image scaling with reduced hardware complexity. For analyzing the performance of the proposed work, two recently developed image scaling techniques [7, 41] are explained in the following section.

## 2. Existing Techniques

**Figure 1:**
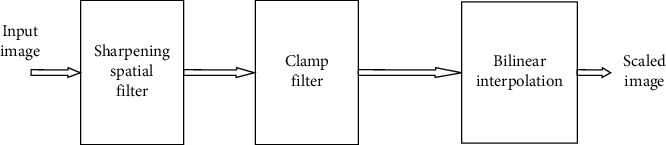
Block diagram of the bilinear interpolation-based image scaling processor.

A low-cost high-quality image scaling processor has been recently proposed [41]. It consists of a sharpening spatial filter, clamp filter, and bilinear interpolation.  Figure 1 shows the block diagram of the bilinear interpolation-based image scaling processor.

The combined sharpening and clamp filters serve as the prefilter to reduce blurring and aliasing artifacts in the scaled image. Hence, the computing resources and memory buffers are reduced by using this technique [41]. The clamp filter, a low-pass filter, is combined with the sharpening spatial filter as the prefilter to reduce the blurring effect. For efficient hardware implementation, a 3 × 3 clamp filter and 3 × 3 sharpening filter are combined together into a 5 × 5 filter as

(1) $$\begin{array}{c} \text{kernel }\left[\text{prefilter}\right]=\frac{\left[\begin{array}{ccc} 1 & 1 & 1\\ 1 & C & 1\\ 1 & 1 & 1 \end{array}\right]}{\left(C+8\right)}*\frac{\left[\begin{array}{ccc} 1 & 1 & 1\\ 1 & S & 1\\ 1 & 1 & 1 \end{array}\right]}{\left(S+8\right)}, \end{array}$$

where *C* is the clamp parameter used to enhance the differences along the direction of edges to reduce the unwanted discontinuous edges and aliasing effects and *S* is the sharp parameter which is used to vary the degree of sharpening. These parameters are set according to the characteristics of the image. The kernel of the combined filter is given as

(2) $$\begin{array}{c} \left|\begin{array}{ccccc} -1 & -2 & -3 & -2 & -1\\ -2 & -2-C+S & -4-C+S & -2-C+S & -2\\ -3 & 4-C+S & -8+SC & 4-C+S & -3\\ -2 & -2-C+S & 4-C+S & -2-C+S & -2\\ -1 & -2 & -3 & -2 & -1 \end{array}\right|. \end{array}$$

**Figure 2:**
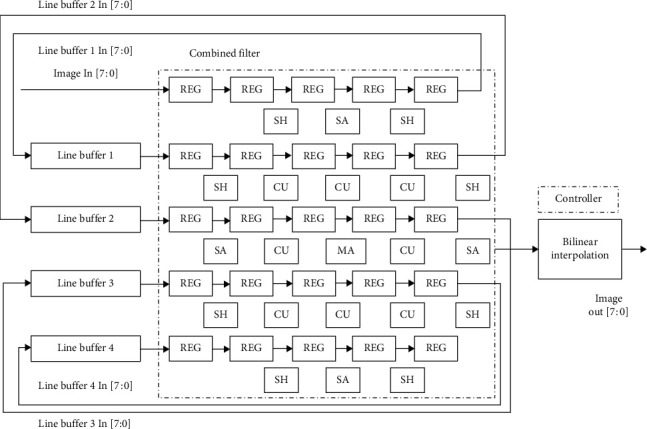
VLSI implementation of the prefilter.

Figure 2 shows the low-cost VLSI implementation of the prefilter for the local window of size 5 × 5 [6, 41]. It consists of 25 shift registers to store 25 pixels of the 5 × 5 local window which is convolved with the coefficients of the prefilter. The convolution operation needs 8 shifters (SH), 5 shifter-adders (SA), 8 calculation units (CU), 1 multiplier-adder (MA), and 24 adders. The calculating unit is designed with a reconfigurable feature for computing clamp and sharp parameters.

The main limitation of this technique is high complexity due to combined filter design, logic for implementing hardware sharing, and reconfigurable techniques.

Another recent technique, a nonadaptive image scaling algorithm, using high-boost filtering has been proposed [7]. The image scaling is performed by linear interpolation, and then enhancement is done using high-boost filtering.

**Figure 3:**
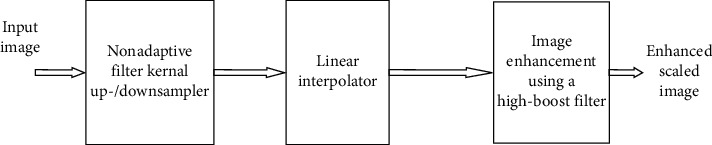
Block diagram of nonadaptive linear interpolation-based image scaling.

This technique results in high-quality image scaling, but the VLSI implementation needs complex hardware. Though many efficient image scaling algorithms have been developed, additional processing is required to enhance the scaling performance. Hence, the main focus of this work is to develop an efficient image scaling algorithm with less hardware complexity.  Figure 3 shows the block diagram of nonadaptive linear interpolation-based image scaling.

For real-time applications, another VLSI architecture is implemented by using the anisotropic probabilistic neural network (APNN) [42]. APNN is one of the interpolation techniques employed within the VLSI architecture that sharply improves the edge region and greatly reduces the blurred effect of an image. In this implementation, the processing speed is usually four times faster than that of the personal computer at 3.4 GHz. However, a huge amount of resource utilization is one of the shortcomings made in the hardware APNN. To save hardware resource utilization, a new approach of the VLSI system is proposed based on unified textual and dynamic compressive features (UTDCF) [43]. It performs several paradigms of memory-centric levels, multiple pipelines, and processing circuits to attain a high frame rate of object tracking capability. This approach not only consumes fewer resources and high speed but also attains reasonable memory consumption. Obviously, these massive parallel circuits attain greater advantages in terms of real-time performance. However, it cannot compete with the majority of embedded applications.

## 3. Proposed Work

### 3.1. Motivation

**Figure 4:**
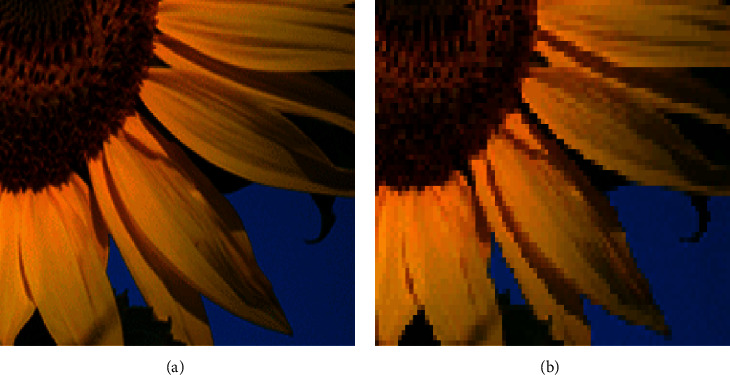
Scaled images in low and high resolutions. (a) 3 megapixels. (b) 1 megapixel.

Image scaling operation enlarges or reduces the size of the image (spatial resolution) in terms of pixels. Image resolution refers to the amount of information an image can hold and is controlled by the number of pixels or bit depth/pixel. As the resolution of an image changes from the capturing device to display or to print device, image scaling is normally required, especially when to match low-resolution display devices to high-resolution devices, and vice versa. When the resolution is larger, the scaling (enlarging) can be possible without any loss of sharpness and image details.  Figure 4 shows the enlarged version of the images captured with different resolutions and squaring effects found in edges of the low-resolution image. Also, the scaled image needs larger memory and longer processing time. Hence, the main focus of this work is to develop a memory-efficient, high-performance VLSI architecture for image scaling algorithm.

### 3.2. Effective Weighted Median Interpolation-Based Image Scaling

**Figure 5:**
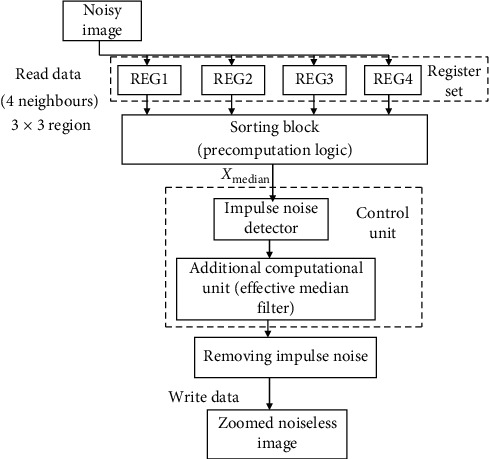
Block diagram of the modified image scaling algorithm.

In this work, an effective weighted median interpolation- (EWMI-) based image scaling algorithm is developed. It is a nonlinear method which performs interpolation as well as denoising. Hence, a low-cost hardware architecture is implemented, and it results in scaled images of high visual quality without using any prefiltering compared to linear interpolation methods.  Figure 5 shows the block diagram of the proposed EWMI image scaling architecture. The major blocks are the register set to hold four neighbours, sorting block, and impulse noise detector and remover blocks. For computing the effective weighted median value for interpolation and denoising, a 3 × 3 local window is considered. An efficient sorter architecture is designed with two features such as precomputation logic and low computation complexity. Precomputation logic is added for power saving, and the sorted array size is made to odd for selecting the median value without addition and division operations.

**Figure 6:**
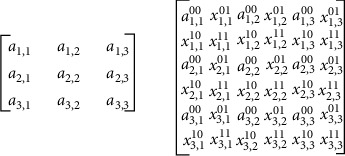
Scaled array with labelled elements.

For image scaling, first, an empty array of size 2*N* × 2*N*, where *N* × *N* is the input image size, is constructed and stored in memory. The array elements are labelled as shown in Figure 6. The elements with label *a*_*i*,*j*_^00^ are replaced by the original pixel values.

**Figure 7:**
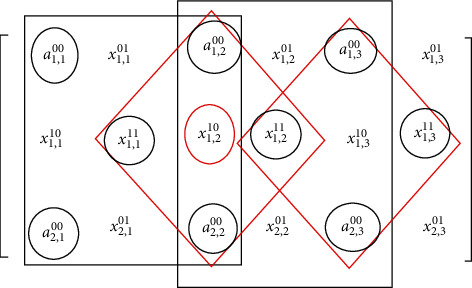
Pipelined processing of successive interpolations.

The remaining elements are interpolated using the proposed EWMI image scaling algorithm. Next, the elements with label *x*_*i*,*j*_^11^ are taken for interpolation. Its four diagonal neighbours (original pixel values) in the local window are multiplied with a weight value of 1 and given to the sorter unit. The median is computed from the sorted array, and then the effective median value is computed to replace *x*_*i*,*j*_^11^. Next, elements with labels *x*_*i*,*j*_^10^ and *x*_*i*,*j*_^01^ are interpolated by considering two horizontal neighbours (original pixels) and two vertical neighbours (previously interpolated pixels). The neighbours with interpolated values are assigned with a suitable weight value in the range of 0.2 to 0.9. Pixels in the scaled array are interpolated by taking diagonal or vertical and horizontal neighbours in a local window of size 3 × 3 as shown in Figure 7. In order to speed up this processing, interpolation using diagonal neighbours can be overlapped with interpolation using vertical and horizontal neighbours using the pipelining technique.

The sorter unit contains binary comparator and swap units. Bitwise comparisons are performed, and the precomputation logic is used to avoid unnecessary switching. Significant power reduction is achieved with negligible area overhead. Sorted array *X* is with four elements, and two centre values are to be added and divided by 2 for computing the median value. To reduce the hardware complexity, the maximum value or minimum value of the sorted array is duplicated to make the array size as 5. *X*(3) is now chosen as the median value for Xmed extra computations. Next, *X*_med_ is tested whether it is a noisy pixel (equal to 0 or 255) or not. If not, *X*_eff_ = *X*_med_; otherwise, *X*_eff_ suitable representative value is computed as per the proposed algorithm.

The effective median value is used for interpolation; also, the relative distance between the pixels in the local window is computed, and the elements which are deviating much from the distance are also identified and replaced by *X*_eff_. Hence, the proposed EWM computation not only performs scaling but also is used for detecting and removing the impulse noise.  Figure 5 represents the modified image scaling algorithm.

### 3.3. EWMI Algorithm

Step 1: given input image of size *N* × *N*, construct an array of size 2*N* × 2*N*
Step 2: label array elements in pixel positions as *a*_*i*,*j*_^00^, *x*_*i*,*j*_^01^, *x*_*i*,*j*_^10^, *x*_*i*,*j*_^11^, where *i*, *j* = 1 to 3, as shown in Figure 7
Step 3: replace elements labelled *a*_*i*,*j*_^00^ by the respective original pixel values
Step 4: define a 3 × 3 local window for each array element *x*_*i*,*j*_^11^, and perform the following:
  - Read and sort the four diagonal neighbours in an ascending order
  - Duplicate the max value into sorted array *X* to make the array size as *X* [5]
  - Select the median value as the centre value *X* = *X*_med_ [3]
  - Define impulse noise values *I*1 = 0 and *I*2 = 255
  - If *X*_med_ ≠ *I*1 or *X*_med_ ≠ *I*2, then *X*_eff_ = *X*_med_, and go to “(g)”
  - Else, compute *X*_eff_ = (*I*1 + I2)/4
  - Find the relative difference between adjacent pixels (*d*_*i*_ = *X*_*i*_ + 2 − *X*_*i*_ + 1) and choose *D*_max_ = max(*d*_*i*_)
  - Replace *x*_*i*,*j*_^11^ by *X*_eff_ and its diagonal neighbours whose *D*_*i*_ > *D*_max_
Step 5: define a 3 × 3 local window for each array element *x*_*i*,*j*_^10^ and
  - Apply the scale value of 0.6 to two horizontal neighbours
  - Read and sort the two horizontal and two vertical neighbours in an ascending order
  - Repeat steps from 4 (b) to 4 (h)
Step 6: define a 3 × 3 window for each array element *x*_*i*,*j*_^10^*x*_*i*,*j*_^01^ and
  - Apply the scale value of 0.6 to two horizontal and two vertical neighbours
  - Read and sort the eight neighbours in an ascending order
  - Repeat steps from 4 (b) to 4 (h).

Using the proposed EWMI image scaling algorithm, interpolation and denoising can be done simultaneously to produce high-quality scaled images. For low-resolution images and images with blurring, defocusing and noise can be scaled with high visual quality using the proposed algorithm.

## 4. Simulation Results and Performance Analysis

Xilinx ISE design suite 13.2 tool has been used for implementing the VLSI architecture of the proposed effective WMI image scaling algorithm using Verilog HDL, and the MATLAB R2010b image processing tool box is used to verify the visual quality of the scaled images. The performance of scaled images of the proposed and existing techniques [7, 41–44] is analyzed in terms of PSNR. Image samples from the LIVE image quality database and real-time blur image database [45, 46] are used for the performance analysis. The real-time blur image database [46] contains 585 images with resolutions ranging from 1280 × 960 to 2272 × 1704 pixels. In this work, based on the resolution, two types of image samples with high- and low-resolution ranges of different sizes are taken, and scaling is performed.

**Figure 8:**
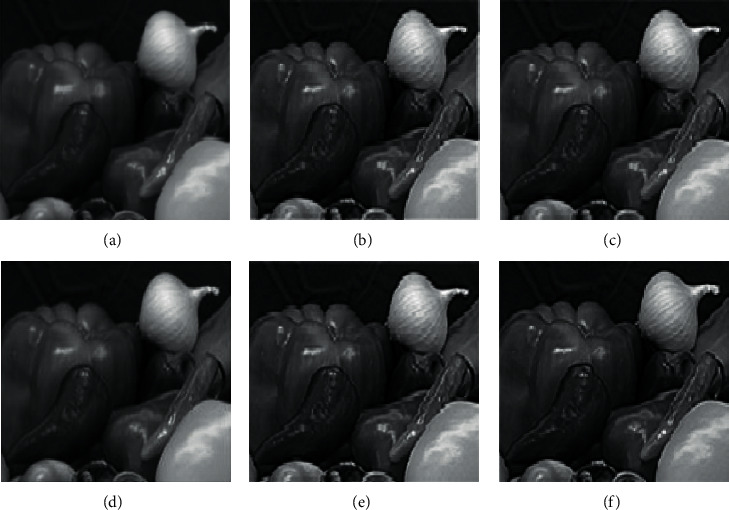
Comparison of existing and proposed techniques with degree of scaling 2 using digital natural images. (a) Bilinear interpolation [44]. (b) BI with the prefilter [41]. (c) High-boost filter [7]. (d) APNN [42]. (e) UTDCF [43]. (f) Proposed EWMI.

**Figure 9:**
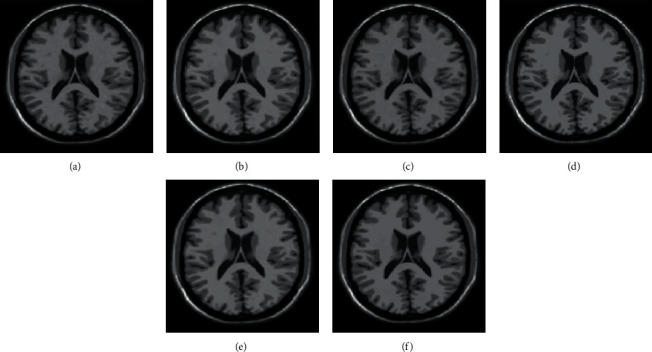
Various techniques are comparatively analyzed by using the digital MR image. (a) Bilinear interpolation [44]. (b) BI with the prefilter [41]. (c) High-boost filter [7]. (d) APNN [42]. (e) UTDCF [43]. (f) Proposed EWMI.

A high-resolution image of size 256 × 256 is scaled into 512 × 512. From Figures 8 and 9, it is confirmed that the edge details are well preserved and better in the proposed than the existing algorithms for both digital natural images and magnetic resonance (MR) image (medical data).

Table 1 represents the comparison of the PSNR value of existing and proposed image scaling techniques with the scale factor as 2.  Table 2 highlights the test sample images.  Table 3 describes the comparison of computing resources and memory requirement.

**Figure 10:**
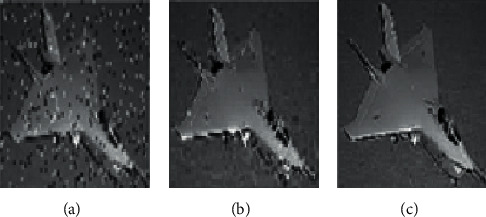
Comparison of existing and proposed techniques with degree of scaling 2. (a) Noisy image. (b) BI with the prefilter. (c) Proposed EWMI.

Table 1 gives PSNR values of scaled images. Some of the sample images from the LIVE image quality database [45] and real-time blur database [46] are given in Table 2. From Table 1, the proposed technique results have better quality (PSNR) than than the existing algorithms for various resolutions, and the artifacts are effectively removed by the proposed algorithm. The performance is even better for noisy images.  Figure 10 shows the image of size 128 × 128 with 0.4 impulse noise and the scaled images by existing and proposed scaling algorithms.

Table 3 gives details of computing resources used for the implementation of existing [41] and proposed architectures for image scaling. It is found that the proposed EWMI image scaling architecture is of low cost compared to the existing one [41]. It inherently removes the noise and is used for scaling operations, whereas in the existing techniques, separate filters are needed to preserve edge details. Hence, the proposed architecture is a low-cost, memory-efficient, and high-quality image scaling algorithm.

## 5. Conclusion

In this work, VLSI implementation of an effective WMI image scaling algorithm is proposed. The main contribution of this work is developing an effective weighted median technique capable of performing interpolation as well as denoising. As the degree of scaling increases, the proposed technique removes the blurring and preserving edge details compared to other existing techniques. The proposed work yields better performance with reasonable hardware complexity. In future, techniques to minimize the hardware complexity of effective WMI image scaling will be performed. The major limitation of the proposed image scaling algorithm is that it is used only for zooming and for scale down, and proper modifications will be done.

## Figures and Tables

**Table 1:** Comparison of PSNR of existing and proposed image scaling techniques with scaling factor 2.

Image details	BI [44]	BI with the prefilter [41]	High-boost filter [7]	APNN [42]	UTDCF [43]	Proposed EWMI
		High-resolution images (4 megapixels)				
Parrots	28.70	39.86	45.94	46.56	47.32	48.39
Flower	27.66	39.45	46.57	46.86	47.92	49.13
Vegetables	29.24	39.58	46.46	47.21	47.56	48.47
		Low-resolution images (1 megapixel)				
Pepper	25.78	31.98	41.23	42.65	43.12	45.44
Camera man	25.93	32.34	40.74	43.23	44.19	45.67
Parrots	26.14	33.56	41.32	42.87	45.54	46.46
		Images with impulse noise (0.4–0.7 variance)				
Parrots	22.45	28.34	39.11	40.04	41.38	42.33
Missile	22.11	28.45	38.76	39.56	42.95	43.67
Lena	21.67	27.99	39.10	40.17	41.93	43.56
		Images with JPEG artifacts and blur				
Parrots	19.75	25.61	36.23	38.54	39.80	41.23
Flower	20.44	25.45	36.79	37.76	38.79	40.74
Vegetables	20.69	26.77	37.21	39.98	40.85	41.32

**Table 2:** Test image samples used for performance analysis.

High resolution (4 megapixels)	Low resolution (1 megapixel)	Image with impulse noise	Image with JPEG artifacts and blur
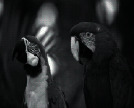	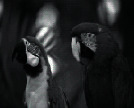	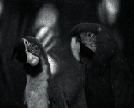	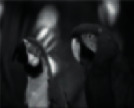
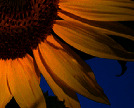	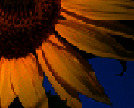	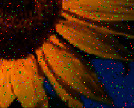	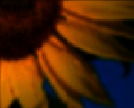
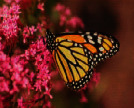	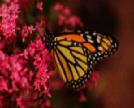	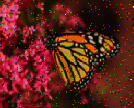	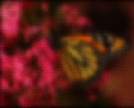

**Table 3:** Comparison of computing resources and memory requirement.

Computing resources	Existing (BI with the prefilter) [41]	Proposed effective WMI
Registers	25	4
Multipliers	5	—
Adder and subtractor	35	1
Shifters	17	1
Comparator and swap units	—	5
Multiplexer	2	—
Line buffers	4	—

## Data Availability

The data that support the findings of this study are available within the article.

## References

[1] Chun-Ho Kim C. H., Si-Mun Seong S. M., Jin-Aeon Lee J. A., Lee-Sup Kim L. S. (2003). Winscale: an image-scaling algorithm using an area pixel model. *IEEE Transactions on Circuits and Systems for Video Technology*.

[2] Jain S., Suresh D. (2013). A new Jpeg image scaling algorithm based on the area pixel model. *International Journal of Engineering Science*.

[3] Hakran Kim H., Youngjoon Cha Y., Seongjai Kim S. (2011). Curvature interpolation method for image zooming. *IEEE Transactions on Image Processing*.

[4] Lin C. C., Sheu M. H., Chiang H. K., Tsai W. K., Wu Z. C. Real-time FPGA architecture of extended linear convolution for digital image scaling.

[5] Lin C. C., Sheu M. H., Chiang H. K., Liaw C., Wu Z. C. The efficient VLSI design of BI-CUBIC convolution interpolation for digital image processing.

[6] Shih-Lun Chen S. L., Hong-Yi Huang H. Y., Ching-Hsing Luo C. H. (2011). A low-cost high-quality adaptive scalar for real-time multimedia applications. *IEEE Transactions on Circuits and Systems for Video Technology*.

[7] Safinaz S. (2014). An efficient algorithm for image scaling with high boost filtering. *International Journal of Scientific and Research Publications*.

[8] Wisesa O., Adriansyah A., Khalaf O. I. Prediction analysis sales for corporate services Telecommunications company using gradient boost algorithm.

[9] Wang X., Liu J., Ibrahim Khalaf O., Liu Z. (2021). Remote sensing monitoring method based on BDS-based maritime joint positioning model. *Computer Modeling in Engineering and Sciences*.

[10] Khalaf O. I. (2021). Preface: smart solutions in mathematical engineering and sciences theory. *Mathematics in Engineering, Science and Aerospace*.

[11] Wisesa O., Andriansyah A., Ibrahim Khalaf O. (2020). Prediction analysis for business to business (B2B) sales of telecommunication services using machine learning techniques. *Majlesi Journal of Electrical Engineering*.

[12] Sengan S., Vidya Sagar P., Ibrahim Khalaf O., Dhanapal R. (2021). The optimization of reconfigured real-time datasets for improving classification performance of machine learning algorithms. *Mathematics in Engineering, Science and Aerospace (MESA)*.

[13] Xiang X., Li Q., Khan S., Khalaf O. I. (2021). Urban water resource management for sustainable environment planning using artificial intelligence techniques. *Environmental Impact Assessment Review*.

[14] Suryanarayana G., Chandran K., Khalaf O. I., Alotaibi Y., Alsufyani A., Alghamdi S. A. (2021). Accurate magnetic resonance image super-resolution using deep networks and Gaussian filtering in the stationary wavelet domain. *IEEE Access*.

[15] Li G., Liu F., Sharma A. (2021). Research on the natural language recognition method based on cluster Analysis using neural network. *Mathematical Problems in Engineering*.

[16] Dalal S., Khalaf O. I. (2021). Prediction of occupation stress by implementing convolutional neural network techniques. *Journal of Cases on Information Technology*.

[17] Sengan S., Koteswara Rao G. R., Ibrahim Khalaf O., Rajesh Babu M. (2021). Markov mathematical analysis for comprehensive real-time data-driven in healthcare. *Mathematics in Engineering, Science and Aerospace (MESA)*.

[18] Tuan Hoang A., Phuong Nguyen X., Ibrahim Khalaf O. (2021). Thermodynamic simulation on the change in phase for carburizing process. *Computers, Materials & Continua*.

[19] Tavera Romero C. A., Castro D. F., Ortiz J. H., Khalaf O. I., Vargas M. A. (2021). Synergy between circular economy and industry 4.0: a literature review. *Sustainability*.

[20] Xuan Tran T., Phuong Nguyen X., Nam Nguyen D. (2021). Effect of poly-alkylene-glycol quenchant on the distortion, hardness, and microstructure of 65Mn steel. *Computers, Materials & Continua*.

[21] Rajasoundaran S., Prabu A. V., Subrahmanyam J. B. V. (2021). Secure watchdog selection using intelligent key management in wireless sensor networks. *Materials Today: Proceedings*.

[22] Tavera C. A., Ortiz J. H., Khalaf O. I., Saavedra D. F., Aldhyani T. H. H. (2021). Wearable wireless body area networks for medical applications. *Computational and Mathematical Methods in Medicine*.

[23] Abdulsahib G. M., Khalaf O. I. (2021). An improved cross-layer proactive congestion in wireless networks. *International Journal of Advances in Soft Computing and Its Applications*.

[24] Abdulsahib G. M., Khalaf O. I. (2021). Accurate and effective data collection with minimum energy path selection in wireless sensor networks using mobile sinks. *Journal of Information Technology Management*.

[25] Khalaf O. I., Abdulsahib G. M. (2021). Optimized dynamic storage of data (ODSD) in IoT based on blockchain for wireless sensor networks. *Peer-to-Peer Networking and Applications*.

[26] Subahi A. F., Alotaibi Y., Khalaf O. I., Ajesh F. (2020). Packet drop battling mechanism for energy aware detection in wireless networks. *Computers, Materials & Continua*.

[27] Khalaf O. I., Abdulsahib G. M. (2020). Energy efficient routing and reliable data transmission protocol in WSN. *International Journal of Advances in Soft Computing and Its Applications*.

[28] Khalaf O. I., Abdulsahib G. M., Sabbar B. M. (2020). Optimization of wireless sensor network coverage using the bee algorithm. *Journal of Information Science and Engineering*.

[29] Khalaf O. I., Ogudo K. A., Singh M. A. (2021). Fuzzy-based optimization technique for the energy and spectrum efficiencies trade-off in cognitive radio-enabled 5G network. *Symmetry*.

[30] Khalaf O. I., Ajesh F., Hamad A. A., Nguyen G. N., Le D.-N. (2020). Efficient dual-cooperative bait detection scheme for collaborative attackers on mobile ad-hoc networks. *IEEE Access*.

[31] Abdullah Hamad A., Al-Obeidi A. S., Al-Taiy E. H., Ibrahim Khalaf O., Le D. (2021). Synchronization phenomena investigation of A new nonlinear dynamical system 4-D by gardano’s and lyapunov’s methods. *Computers, Materials & Continua*.

[32] Ibrahim Khalaf O., Sokiyna M., Alotaibi Y., Alsufyani A., Alghamdi S. (2021). Web attack detection using the input validation method: dpda theory. *Computers, Materials & Continua*.

[33] T Romero C. A., Ortiz J. H., Khalaf O. I., Prado A. R. (2021). Web application commercial design for financial entities based on business intelligence. *Computers, Materials & Continua*.

[34] A.R. Alkhafaji A., Nur Amir Sjarif N., Shahidan M. A. (2021). Payload capacity scheme for quran text watermarking based on vowels with kashida. *Computers, Materials & Continua*.

[35] Prasad S. K., Rachna J., Khalaf O. I., Le D.-N. (2020). Map matching algorithm: real time location tracking for smart security application. *Telecommunications and Radio Engineering*.

[36] Nabiel Al-Khanak E., Peck Lee S., Ur Rehman Khan S. (2021). A heuristics-based cost model for scientific workflow scheduling in cloud. *Computers, Materials & Continua*.

[37] Zhao H., Chen P. L., Khan S., Khalafe O. I. (2020). Research on the optimization of the management process on internet of things (IoT) for electronic market. *The Electronic Library*.

[38] Krichen M., Mechti S., Alroobaea R. (2021). A formal testing model for operating room control system using internet of things. *Computers, Materials & Continua*.

[39] Javed Awan M., Shafry Mohd Rahim M., Nobanee H., Yasin A., Ibrahim Khalaf O., Ishfaq U. (2021). A big data approach to black Friday sales. *Intelligent Automation & Soft Computing*.

[40] Zheng X., Ping F., Pu Y., Wang Y., Montenegro-Marin C. E., Khalaf O. I. (2021). Recognize and regulate the importance of work-place emotions based on organizational adaptive emotion control. *Aggression and Violent Behavior*.

[41] Chen S.-L. (2013). VLSI implementation of a low-cost high-quality image scaling processor. *IEEE Transactions on circuits and systems, express briefs*.

[42] Chen C.-H., Chang H.-W., Kuo C.-M. (2019). VLSI implementation of anisotropic probabilistic neural network for real-time image scaling. *Journal of Real-Time Image Processing*.

[43] He W., Zhang J., Lin Y. (2020). A low-cost high-speed object tracking VLSI system based on unified textural and dynamic compressive features. *IEEE Transactions on Circuits and Systems II: Express Briefs*.

[44] Dilip P. A., Rameshbabu K., Ashok K. P., Shivdas S. A. (2014). Bilinear interpolation image scaling processor for VLSI architecture. *International Journal of Reconfigurable and Embedded Systems*.

[45] Sheikh H. R., Wang Z., Cormack L. K., Bovik A. C. LIVE image quality assessment database release 2. http://live.ece.utexas.edu/research/quality/subjective.

[46] BID–blurred image database. http://www.lps.ufrj.br/profs/eduardo/.

